# Pediatric Avoidant-Restrictive Food Intake Disorder and gastrointestinal-related Somatic Symptom Disorders: Overlap in clinical presentation

**DOI:** 10.1177/13591045211048170

**Published:** 2021-11-13

**Authors:** Katelynn E Boerner, Jennifer S Coelho, Fiza Syal, Deepika Bajaj, Natalie Finner, Amrit K Dhariwal

**Affiliations:** 1 Department of Pediatrics, 12358 BC Children’s Hospital Research Institute and University of British Columbia, Vancouver, BC, Canada; 2 Provincial Specialized Eating Disorders Program for Children & Adolescents, BC Children’s Hospital, Vancouver, BC, Canada; 3 Department of Psychiatry, 8166 University of British Columbia, Vancouver, BC, Canada; 4 Division of Adolescent Medicine, 27338 Children’s Hospital of Eastern Ontario, Ottawa, ON, Canada; 5 Department of Psychiatry, 37210 BC Children’s Hospital, Vancouver, BC, Canada

**Keywords:** Avoidant-Restrictive Food Intake Disorder, feeding and eating disorders, functional gastrointestinal disorders, adolescent health, somatoform, disorders, somatization disorder, comorbidity, child psychiatry, adolescent psychiatry, medical psychology

## Abstract

Certain presentations of Avoidant/Restrictive Food Intake Disorder (ARFID) and Somatic Symptom and Related Disorders (SSRDs) have conceptual overlap, namely, distress and impairment related to a physical symptom. This study compared characteristics of pediatric patients diagnosed with ARFID to those with gastrointestinal (GI)-related SSRD. A 5-year retrospective chart review at a tertiary care pediatric hospital comparing assessment data of patients with a diagnosis of ARFID (*n* = 62; 69% girls, *M*_age_ = 14.08 years) or a GI-related SSRD (*n* = 37; 68% girls, *M*_age_ = 14.25 years). Patients diagnosed with ARFID had a significantly lower percentage of median BMI than those with GI-related SSRD. Patients diagnosed with ARFID were most often assessed in the Eating Disorders Program, whereas patients diagnosed with an SSRD were most often assessed by Consultation-Liaison Psychiatry. Groups did not differ on demographics, psychiatric diagnoses, illness duration, or pre-assessment services/medications. GI symptoms were common across groups. Patients diagnosed with an SSRD had more co-occurring medical diagnoses. A subset (16%) of patients reported symptoms consistent with both diagnoses. Overlap is observed in the clinical presentation of pediatric patients diagnosed with ARFID or GI-related SSRD. Some group differences emerged, including anthropometric measurements and co-occurring medical conditions. Findings may inform diagnostic classification and treatment approach.

## Introduction

Historically, there has been a subset of individuals with a clinically significant eating condition for whom the diagnosis of a specified eating disorder (i.e., anorexia nervosa or bulimia nervosa) was not appropriate, because the youth did not report body image concerns. While the DSM-IV-TR (American Psychiatric Association, 2000) diagnosis of “feeding disorder of infancy or early childhood” was appropriate for young children with an onset of feeding problems before the age of 6, there was no diagnosis relevant for those with a later age of onset. With the DSM-5 (American Psychiatric Association, 2013), Avoidant/Restrictive Food Intake Disorder (ARFID) was introduced to capture restrictive eating concerns across the lifespan. Avoidant/Restrictive Food Intake Disorder is a clinically significant eating disturbance that has serious consequences (e.g., weight loss or failure to make expected weight gain, nutritional deficiency, and psychosocial impairment) but is not associated with a preoccupation with weight or shape.

The majority of the literature on ARFID has been published by specialized eating disorder services (Bourne et al., 2020). Compared to patients with other eating disorders (EDs), patients with ARFID are more commonly: younger, male, with a comorbid medical condition or anxiety disorder, and present with selective eating, anxiety, and/or gastrointestinal (GI) symptoms (Fisher et al., 2014). Some of these characteristics suggest an overlap between symptoms of ARFID and Somatic Symptom and Related Disorders (SSRD). Specifically, there is a subset of patients with ARFID (∼43%) who fear aversive consequences of eating, commonly GI symptoms such as abdominal pain, dysphagia, heartburn, or nausea (Cooney et al., 2018; Norris et al., 2018) or related to Disorders of Gut–Brain Interaction (Murray et al., 2020). Furthermore, many patients with ARFID describe other physical sensations (e.g., chest pain) or related worries (e.g., fear of vomiting or choking and aversion to food textures) that may also be associated with a general heightened awareness of somatic sensations (Fisher et al., 2014; Norris et al., 2014; Stein et al., 2021). SSRDs represent a related condition in which individuals present with worries about aversive somatic experiences, but in the absence of identified problems with eating (though the extent to which eating disturbances arise in SSRDs is not well documented). As such, ARFID and SSRDs may share common etiological pathways.

Patients with SSRDs experience physical symptoms that are distressing and/or significantly impact their daily functioning. Previous literature describes a period of “confusion” prior to receiving a diagnosis of an SSRD, whereby patients and families undergo numerous consultations and investigations (Dhariwal et al., 2017). However, once the diagnosis is conferred, there are established clinical pathways (Ibeziako et al., 2019) and evidence-based treatments (Bonvanie et al., 2017). Such treatments may be appropriate for adaptation to an ARFID treatment context for youth whose eating disturbances are associated with a distressing somatic symptom.

A retrospective chart review comparing pediatric patients with SSRD and eating disorders found that patients with EDs had longer medical admissions, more depressive disorders, suicidal ideation, and self-harm, whereas patients with SSRDs had greater utilization of emergency department and hospital services, and higher rates of learning difficulties and trauma (Ibeziako et al., 2016). However, no study to date has compared SSRDs to ARFID specifically, differentiated from other eating disorder subtypes. Anxiety disorders are commonly reported as co-occurring diagnoses for both SSRDs and ARFID. Depression frequently co-occurs in patients with an SSRD (Bujoreanu et al., 2014), whereas neurodevelopmental disorders (specifically Autism Spectrum Disorder) commonly co-occur in patients diagnosed with ARFID (Inoue et al., 2021).

### Rationale, aims, and hypotheses for the current study

Existing research on ARFID presentations suggest a conceptual overlap with SSRDs, at least for a subset of patients. The literature on GI-related somatic symptoms, such as functional nausea, has also described patients engaging in food restriction in an attempt to manage their symptoms, with associated weight loss (Cole et al., 2020). However to our knowledge, no research has examined the overlap of ARFID with SSRDs. We describe a retrospective chart review providing preliminary data to guide further inquiry.

The current study compared patients diagnosed with ARFID to patients diagnosed with an SSRD with predominant GI symptoms. The proportion of patients who met criteria for both diagnoses was examined, as an overlap across diagnoses was expected. Commensurate with the diagnostic criteria for each disorder, patients with GI-related SSRDs were expected to present with more physical symptoms, and patients with ARFID were expected to present with lower percent of expected body weight. A high prevalence of co-occurring anxiety disorders was expected in both groups, with more mood disorders in the SSRD group, and more neurodevelopmental disorders in the ARFID group. Patients with a GI-related SSRD were expected to have a longer illness duration and more treatment services prior to assessment.

## Methods

### Chart identification

A retrospective chart review was conducted at a tertiary-level pediatric hospital in Western Canada. This hospital has a specialized eating disorders program that offers clinical care for children and adolescents in outpatient, day treatment, and inpatient settings (Coelho et al., 2018). Patients with a diagnosis of ARFID are also seen through other services in the hospital, including Gastroenterology and Feeding Clinic, Medical Psychology, and Consultation-Liaison Psychiatry. Patients with a diagnosis of a GI-related SSRD are similarly dispersed across hospital services, with a specialized group treatment for SSRDs within the Department of Psychiatry (Mind-Body Connection Program) (Dhariwal et al., 2017).

Children and youth who were assessed and/or admitted during the 5-year study period (January 1, 2014 to January 1, 2019) and were assigned a diagnosis of ARFID or a GI-related SSRD were included in this study. Ethical approval was obtained by the University of British Columbia Children’s and Women’s Clinical Research Ethics Board (H18-03435).^1^ See Supplementary Materials for details on chart identification.^2^ Potentially eligible medical records were reviewed by the research team, with 10.5% (99 of 940) meeting inclusion criteria.

### Data extraction

Medical records were examined by a member of the research team, who extracted data according to a standardized manual. Data was extracted into Research Electronic Data Capture tools hosted at BC Children’s Hospital Research Institute (Harris et al., 2009). Data extracted included general demographics, anthropometric data, physical symptoms, psychiatric diagnoses, trauma history, substance use, history of self-harm or suicidality, illness duration, and treatments accessed. Data were checked by another member of the research team. Discrepancies or questions about data entry that could not be resolved with the data extraction manual were reviewed by a senior member of the research team (JSC).

Participants were identified as belonging to one of two groups based on their primary presenting diagnosis: ARFID (*n* = 62) or GI-related SSRD (*n* = 37); see Supplementary Materials for more information. Additional sub-analyses were conducted to characterize individuals who had symptoms of both diagnoses.

### Data analysis

A power calculation conducted with G*Power 3.1 (Faul et al., 2007) determined that a sample size of 19 and 39 per group with a 2:1 allocation ratio was sufficient to detect a large effect size with a two-tailed *t*-test, at *α* = .05 and power = 0.8. For the chi-square analyses at *α* = .05 and power = 0.8 for two groups, a total sample size of 88 was required to detect a medium effect size.

Data were analyzed in SPSS 24.0. Chi-square analyses (for categorical variables) and two-tailed independent sample *t*-tests (for continuous variables) were used to describe group differences. Effect sizes and estimates of precision were calculated for parametric tests. To reduce the number of comparisons, sub-analyses (e.g., comparing the two groups on each individual sub-category, such as specific medication classes under the category of “psychopharmacology”) were only reported if the overall analysis of that category demonstrated a significant difference. A Bonferroni correction was conducted if there were multiple analyses related to a hypothesis (for co-occurring physical/psychiatric symptoms/diagnoses *p* < .05/3 analyses; for services accessed *p* < .05/7 analyses; for specific co-occurring medical conditions *p* < .05/6). Substantial data was missing for the GI-related SSRD group for percent of median BMI (59.5% missing for SSRD sample vs. 2% missing for ARFID sample); available data was interpreted with caution.

## Results

### Diagnostic overlap

Groups were categorized based on the primary diagnosis listed, but it was also noted whether they had features of both diagnoses. There was no significant difference in the number of participants in the ARFID (*n* = 8, 12.9%) and GI-related SSRD (*n* = 8, 21.6%) groups who had both diagnoses, χ^2^(1, *N* = 99) = 1.300, *p* = .254. Note that this comparison included both cases of “full” and “rule-out” diagnoses, as this reflected that there were sufficient symptoms of both diagnoses to indicate clinically significant concerns. Patients with both diagnoses were most often assessed in the Eating Disorders Program or Gastroenterology service.

### Demographic and anthropometric data

Patients diagnosed with ARFID did not differ from patients diagnosed with a GI-related SSRD on age, sex, or gender identity; see Table 1. Patients diagnosed with ARFID had a significantly lower percent of median BMI according to the World Health Organization (WHO) guidelines. Based on a cut-off of <95% of median BMI as an indicator commonly used to denote low weight in eating disorders (Le Grange et al., 2019), 95% (*n* = 58/61) of patients with a diagnosis of ARFID were categorized as low weight, and 73% (*n =* 11/15) of patients with a diagnosis of a GI-related SSRD for whom BMI data were available were categorized as low weight.

**Table 1.** Group differences in demographics, symptom presentation, comorbidities, and psychiatric history (mean and standard deviation or number and percentage, followed by overall sample size).

	ARFID	GI-related SSRD	Difference between ARFID and GI-related SSRD
Age (years)	14.08 (2.88), 62 Range = 5.19–18.23	14.25 (2.31), 37 Range = 9.29–18.00	*t*(97) = −.311, *p* = .756, 95% CI [−.47,.34], *d* = −.06
Sex assigned at birth			
Female	43 (69.4%), 62	25 (67.6%), 37	χ^2^(1, *N* = 99) = .034, *p* = .853
Male	19 (30.6%), 62	12 (32.4%), 37	
Gender identity different than sex			
No	60 (96.8%), 62	36 (97.3%), 37	*p* = 1.000^b^
Yes	2 (3.2%), 62	1 (2.7%), 37	
**Percent of median BMI according to the WHO** ^a^	**80.08% (10.13), 61 Range = 61.6%–107.8%**	**92.75% (18.60), 15 Range = 74.76%–149.75%**	***t*(74) =** −3.605, *p* = .001**, **95% CI [**−1.62**,**-0.44**], *d* =** −1.04
Physical symptoms reported at assessment^c^	58 (93.5%), 62	37 (100%), 37	*p* = .294^b^
Cognitive problems	17 (27.4%)	11 (29.7%)	
Dizziness or fainting/syncope	15 (24.2%)	14 (37.8%)	
Gastrointestinal	52 (83.9%)	37 (100%)	
Headache or migraine	21 (33.9%)	14 (37.8%)	
Movement problems	2 (3.2%)	16 (43.2%)	
Perceptual disturbances	3 (4.8%)	9 (24.3%)	
Sensory problems	8 (12.9%)	9 (24.3%)	
Other pain	12 (19.4%)	15 (40.5%)	
Other symptoms	18 (29%)	1 (2.7%)	
**Co-occurring medical diagnosis** ^c^	**14 (22.6%), 62**	**17 (45.9%), 37**	χ^**2**^(1, *N* = 99) = 5.882, *p* = .015*
Chronic (non-abdominal) pain^d^	2 (3.2%)	4 (10.8%)	*p* = .193^b^
Endocrine conditions^d^	2 (3.2%)	1 (2.7%)	*p* = 1.000^b^
Gastrointestinal conditions^d^	6 (9.7%)	4 (10.8%)	*p* = 1.000^b^
**Neurological conditions**^d^	**1 (1.6%)**	**7 (18.9%)**	***p* = .004** ^b^
Oncological conditions^d^	1 (1.6%)	1 (2.7%)	*p* = 1.000^b^
Other^d^	5 (8.1%)	4 (10.8%)	*p* = .724^b^
Co-occurring psychiatric diagnosis^c,e^	33 (53.2%), 62	19 (51.4%), 37	χ^2^(1, *N* = 99) = .033, *p* = .857
Bipolar-related or depressive disorder	6 (9.7%)	4 (10.8%)	
Anxiety disorder	27 (43.5%)	15 (40.5%)	
Obsessive-compulsive or related disorder	7 (11.3%)	4 (10.8%)	
Traumatic or stressor-related disorder	2 (3.2%)	0 (0%)	
Disruptive, impulse-control, or conduct disorder	1 (1.6%)	1 (2.7%)	
Neurodevelopmental disorder	8 (12.9%)	6 (16.2%)	
History of abuse			
Physical	4 (6.5%), 62	0 (0%), 37	*p* = .294^b^
Sexual	1 (1.6%), 62	3 (8.1%), 37	*p* = .146^b^
Self-harm and suicidality			
History of self-harm	8 (12.9%), 62	7 (18.9%), 37	χ^2^(1, *N* = 99) = .652, *p* = .419
History of suicidal ideation	17 (27.4%), 62	16 (43.2%), 37	χ^2^(1, *N* = 99) = 2.611, *p* = .106
History of suicide attempt	4 (6.5%), 62	2 (5.4%), 37	*p* = 1.000 ^b^
Substance use (historical and/or current)	8 (12.9%), 62	5 (13.5%), 37	*p* = 1.000^b^
Illicit substance use	6 (9.7%)	4 (10.8%)	
Prescription medication misuse	2 (3.2%)	0 (0%)	
Alcohol use	8 (12.9%)	3 (8.1%)	

*Note.* ARFID = Avoidant-Restrictive Food Intake Disorder; CI = confidence interval; GI = gastrointestinal; SSRD = Somatic Symptom and Related Disorder; WHO = World Health Organization.

*p < .05; **p < .01; ***p < .001; analyses reaching significance are bolded.

^a^If data were unavailable at admission/assessment, the height/weight taken at the nearest time point to the assessment was recorded, within 1 month. If not available within 1 month of assessment, recorded as missing data.

^b^Fisher’s exact test (2-sided) used as the assumption of expected frequencies was violated.

^c^Evaluated against a Bonferroni-correlated level of *p* < .017.

^d^Evaluated against a Bonferroni-adjusted level of *p* < .008.

^e^Includes only “full” diagnoses. Data relating to schizophrenia spectrum/psychotic disorder diagnoses were extracted but not included as no participants had a diagnosis in this category.

### Co-occurring physical and psychiatric concerns

Both groups had similar frequencies of physical symptoms reported at assessment, though patients diagnosed with a GI-related SSRD had more co-occurring medical diagnoses (specifically, more neurological conditions); see Table 1. The two groups did not differ significantly on history of abuse, self-harm and suicidality, substance use, or psychiatric diagnoses. In both groups, anxiety disorders were the most common co-occurring psychiatric diagnosis, followed by neurodevelopmental, obsessive-compulsive, and mood disorders. Trauma disorders, disruptive/conduct disorders, and psychotic disorders were rare or absent in both groups.

While the analyses of co-occurring psychiatric disorders examined only “full” diagnoses, secondary exploratory analyses were also conducted for “rule-out” diagnoses of neurodevelopmental disorders, given that more extensive follow-up assessment would have been necessary to confirm the diagnosis if symptoms were present but had not been previously diagnosed. No significant differences were observed in rates of “rule-out” diagnoses for neurodevelopmental disorders (17.7% ARFID (*n* = 11) and 10.8% SSRD (*n* = 4), χ^2^(1, *N* = 99) = .866, *p* = .352; similar results when examining autism specifically).

### Illness duration and healthcare utilization

Table 2 presents group differences in illness duration, healthcare utilization, and treatment prior to assessment. Consultation-Liaison Psychiatry was the most common assessing service for patients diagnosed with a GI-related SSRD, and Eating Disorders Program was the most common assessing service for patients diagnosed with ARFID. There were no significant differences in illness duration, pharmacological medication at the time of assessment, or prior services accessed.

**Table 2.** Group differences in illness duration, healthcare utilization, and treatment data (mean and standard deviation or number and percentage, followed by overall sample size).

	ARFID	GI-related SSRD	Difference between ARFID and GI-related SSRD
**Clinic initially referred to**			*Fisher’s = 55.876, p <* .001*** ^b^
Adolescent Medicine	4 (6.5%), 62	1 (2.7%), 37	
**Consultation-Liaison Psychiatry**	**4 (6.5%), 62**	**26 (70.3%), 37**	
**Eating Disorders**	**40 (64.5%), 62**	**3 (8.1%), 37**	
Gastroenterology	7 (11.3%), 62	3 (8.1%), 37	
Medical Psychology	0 (0%), 62	1 (2.7%), 37	
Mind-Body Connection Program/Outpatient Mental Health Teaching Clinic^a^	1 (1.6%), 62	2 (5.4%), 37	
Other medical/psychiatric subspecialty clinics	6 (9.7%), 62	1 (2.7%), 37	
Pre-assessment illness duration (years)	2.02 (2.77), 59	1.42 (1.80), 35	*t*(92) = 1.148, *p* =.254 95% CI [−.18,.66], *d* = .24
Pharmacological medication at time of assessment	26 (41.9%), 62	16 (43.2%), 37	χ^2^(1, *N* = 99) = .016, *p* = .899
Antidepressant	20 (32.3%)	16 (43.2%)	
Antipsychotic	5 (8.1%)	4 (10.8%)	
Anxiolytic	6 (9.7%)	1 (2.7%)	
Stimulant	2 (3.2%)	0 (0%)	
Other (e.g., anticonvulsant)	0 (0%)	3 (8.1%)	
Services accessed prior to assessment (includes past and current)			
Outpatient services			
Mental health^c^	40 (64.5%), 62	22 (59.5%), 37	χ^2^(1, *N* = 99) = .253, *p* = .615
Physical health (e.g., medical subspecialty clinic)^c^	37 (59.7%), 62	25 (67.9%), 37	χ^2^(1, *N* = 99) = .616, *p* = .432
Community hospital inpatient services			
Mental Health Unit^c^	4 (6.5%), 62	2 (5.4%), 37	*p* = 1.000^b^
Medical Unit^c^	12 (19.4%), 62	7 (18.9%), 37	χ^2^(1, *N* = 99) = .003, *p* = .958
Tertiary inpatient services			
Mental Health Unit^c^	4 (6.5%), 62	4 (10.8%), 37	*p* = .467^b^
Medical Unit^c^	12 (19.4%), 62	10 (27.0%), 37	χ^2^(1, *N* = 99) = .789, *p* = .374
Specialized services for ARFID or SSRD^c^	5 (8.1%), 62	3 (8.1%), 37	*p* = 1.000^b^

*Note.* ARFID = Avoidant-Restrictive Food Intake Disorder; GI = gastrointestinal; SSRD = Somatic Symptom and Related Disorder. Data related to residential and day treatment for eating disorders were extracted, but not included as no participants accessed these services. Participants were also reported to have accessed “other” services, such as the emergency department and alternative medicine practitioners.

*p < .05; **p < .01; ***p < .001; analyses that were significant are bolded.

^a^Mind-Body Connection Program is a group treatment for youth with somatization and their families.

^b^Fisher’s exact test (2-sided) used as the assumption of expected frequencies was violated.

^c^Evaluated against a Bonferroni-adjusted *p* < .007.

## Discussion

There was a great degree of overlap in the clinical presentation of youth with ARFID and GI-related SSRDs. A small subset of the sample had symptoms consistent with co-occurring diagnoses of ARFID and SSRD. As expected, patients with a diagnosis of ARFID had a lower percent of expected body weight. Counter to expectations, patients with a diagnosis of SSRD did not have more physical symptoms but did have more co-occurring medical conditions. There were no differences between diagnostic groups in prevalence of psychiatric disorders, illness duration, or treatments accessed.

One of the goals of the study was to examine diagnostic overlap across these two groups. Only 16% of the total sample reported symptoms consistent with co-occurring diagnoses of ARFID and SSRD. This seeming juxtaposition may be explained considering youth perhaps had features of both conditions but not at a level sufficient to warrant diagnosis (i.e., sub-clinical symptoms). It may also be a reflection of diagnostic practices (i.e., only assigning one “most responsible” diagnosis) or clinician comfort in diagnosing ARFID and/or SSRDs. It is possible that the subgroup of youth who presented with symptoms of both diagnoses represents the aversive-consequences subtype of ARFID, though the features of ARFID were not extracted as part of this chart review (due to lack of use of a validated assessment tool and ambiguity in the clinical notes), so it is not possible to interpret categorization or the dimensional presentation of the individuals in this study.

As expected, patients with a diagnosis of ARFID were at a lower percentage of median BMI than patients with a GI-related SSRD diagnosis. This finding is in line with previous research in youth with Irritable Bowel Syndrome, who reported more alterations to intake/eating behaviors compared to a healthy control group, but no associated change in BMI (Reed-Knight et al., 2016). However, within the GI-related SSRD group the range and *SD* suggests substantial variability, and the majority would be categorized as low weight (i.e., below the median BMI for their age and sex). Additionally, information on BMI was missing from more than half of the patients in the GI-related SSRD group. There may have been systematic differences in patients for whom this information was available in the chart (e.g., weight may have been recorded if there were specific weight- or eating-related concerns noted, or if the patient was receiving a weight-adjusted dosing of medication). Further prospective investigations on the impact of GI symptoms on eating/weight in youth are warranted, utilizing measures that capture eating disturbances beyond body image concerns.

There were no significant demographic differences observed between ARFID and SSRD patient groups. Both samples included a similar preponderance of female patients. The demographics of the present sample are similar to previous chart reviews of individuals with ARFID (Norris et al., 2018) and SSRD (Bujoreanu et al., 2014) from other tertiary-level pediatric hospitals, though these reports described a higher prevalence of mood disorders in SSRD patients than is reported in the current sample.

Both groups had similar frequencies of physical symptoms, though patients diagnosed with SSRD had more co-occurring medical conditions. The high proportion of patients with ARFID reporting GI symptoms (84%) may be relevant to understanding the underlying etiology of certain presentations of this diagnosis that may overlap with GI disorders, including central sensitization (Sim et al., 2021) and interactions of altered gut physiology with symptom-related distress and fear-learning (Nicholas et al., 2021; Wildes et al., 2021). However, it is also possible that some of the reported GI concerns were consequences of malnutrition, such as constipation, rather than an initiating factor for the eating disturbance. In this study, GI symptoms were conceptualized broadly, and included early satiety and upper GI symptoms such as gagging, choking, globus sensation, and dysphagia. Regardless of their role in the onset of ARFID, GI symptoms may serve to maintain avoidant/restrictive eating behaviors. As such, assessing and addressing GI symptoms should be a priority in clinical care for ARFID.

While GI symptoms were the most common type of symptom in each group, movement problems (e.g., tremors, seizures, and weakness) were the second-most common symptoms for the GI-related SSRD sample. This may reflect the fact that neurological conditions (e.g., traumatic brain injury and epileptic and non-epileptic seizures) were the most common co-occurring medical condition for patients with a GI-related SSRD diagnosis, and were significantly more common than in the ARFID patient group. Headache/migraine was the second-most common symptoms within the ARFID patient group, followed by “other” symptoms. The “other” symptoms described in patients with a diagnosis of ARFID were often consistent with a low weight, such as cold intolerance, fatigue, and palpitations. For patients with an ARFID diagnosis, GI conditions (e.g., chronic abdominal pain and inflammatory conditions) were the most common co-occurring medical condition, consistent with chart reviews in adults with a diagnosis of ARFID (Cooper et al., 2021).

Contrary to our hypotheses, there were no differences in the frequency of co-occurring psychiatric disorders between the two groups. A co-occurring psychiatric diagnosis was present in >50% of each group, with anxiety disorders being the most common. The frequency of co-occurring diagnoses in both groups suggests the potential utility of a transdiagnostic treatment approach that targets key maintaining mechanisms of the somatic and/or eating concerns. Reducing the silos between physical and mental health care, and providing a holistic treatment that considers the mind**–**body connection improves clinical care for patients with somatic symptoms (Dhariwal et al., 2017; Jenkins & Smart, 2020), and will likely also benefit patients with ARFID.

The variety of medical and mental health settings across which patients were assessed highlights the importance of provider familiarity with both diagnoses. Additionally, patients with symptoms of both diagnoses were most commonly seen in Eating Disorders and Gastroenterology, again suggesting that there is not a single common pathway for assessment of these conditions and that availability of multi-disciplinary teams to address the complex medical-psychosocial integration inherent in these disorders is critical. In contrast to hypotheses, there were no group differences in illness duration or service utilization prior to assessment. In SSRD treatment, patients and families are characterized as undergoing a series of phases through their illness and treatment journey, including confusion, making connections, integrated treatment, and recovery (Dhariwal et al., 2017). The service utilization for patients with a diagnosis of SSRD likely represents the confusion stage that many families experience of undergoing numerous medical investigations without receiving a definitive answer regarding the cause of their symptoms. The common service utilization across diagnoses may reflect that patients with ARFID undergo a similar period of diagnostic “confusion,” as both disorders are not well-understood and many providers do not feel confident treating them (Coelho et al., 2020; Ibeziako et al., 2019). Similarly, a recent chart review of patients hospitalized for ARFID describes co-occurring anxiety and GI diagnoses with a history of non-revealing investigations and outpatient mental health treatment (Tsang et al., 2020).

As the data being extracted were primarily in the form of free-form clinical notes there may have been discrepancies in the availability of information. This may reflect protocols of specific programs, practices of certain clinicians (e.g., making a definitive diagnosis vs. a “rule-out”), and the focus of the assessment. For example, just as there was significant information missing regarding BMI for patients with a GI-related SSRD, it is possible that complete information about comorbid medical diagnoses and presenting physical symptoms may not have been captured in the medical record for individuals who were being assessed for an ED.

There is a debate in the field about what conditions represent an SSRD, and discrepancies in terminology used to describe such conditions (Boerner et al., 2020). As such, the sample presented may not be representative of all the GI-related SSRD patients who were admitted to the hospital during the study period. Similarly, concerns about the validity of the current diagnostic criteria for ARFID have been raised, particularly in the context of co-occurring psychiatric and medical conditions (Strand et al., 2019). Integration of standardized assessment tools can help clarify clinical presentations of ARFID. The Pica, ARFID, and Rumination Disorders Interview is a structured interview that has promising psychometric properties, and that would provide insight into dimensions of ARFID symptoms across patient populations (Bryant-Waugh et al., 2019). Despite these limitations, chart review remained a logical methodology to use in exploring this emerging area of practice using real-world data to help guide future research.

This exploratory comparative study aimed to provide data regarding the clinical presentation of patients with a diagnosis of ARFID presenting for tertiary care inpatient and/or specialized eating disorders or somatic symptom services, and whether the characteristics of this population overlap with patients diagnosed with a GI-related SSRD. Given that ARFID is a relatively new diagnosis for which treatments are still being established, the substantial overlap in presentation between the two samples suggests the potential clinical utility of adapting established evidence-based treatments for SSRDs for the ARFID population. Further information is needed regarding the similarity across the groups in the primary SSRD treatment targets, such as emotional distress and quality of life. Research has supported ARFID “subtypes” or dimensions (Norris et al., 2018; Thomas et al., 2017). Further exploration of these dimensions may elucidate whether there is a somatically-focused subtype of ARFID that may particularly benefit from SSRD-oriented treatments.

In addition to helping characterize the profile of patients diagnosed with ARFID, the present research reinforces the importance of considering weight and eating-related factors in patients with GI-related somatic symptoms (Stein et al., 2021). Further research is needed to examine the extent to which eating or weight disturbances are problematic in this population, which may necessitate more proactive screening within gastroenterology and somatic symptom assessment settings.

## Supplemental Material

sj-pdf-1-ccp-10.1177_13591045211048170 – Supplemental Material for Pediatric Avoidant-Restrictive Food Intake Disorder and gastrointestinal-related Somatic Symptom Disorders: Overlap in clinical presentation Click here for additional data file. Supplemental Material, sj-pdf-1-ccp-10.1177_13591045211048170 for Pediatric Avoidant-Restrictive Food Intake Disorder and gastrointestinal-related Somatic Symptom Disorders: Overlap in clinical presentation by Katelynn E Boerner, Jennifer S Coelho, Fiza Syal, Deepika Bajaj, Natalie Finner and Amrit K Dhariwal in Clinical Child Psychology and Psychiatry
