# Supplementary material for: Pediatric Avoidant-Restrictive Food Intake Disorder and gastrointestinal-related Somatic Symptom Disorders: Overlap in clinical presentation
Source: Clin Child Psychol Psychiatry. 2021 Nov 13;27(2):385–98. doi: 10.1177/13591045211048170 (PMC9047093; doi:10.1177/13591045211048170)
Supplement: sj-pdf-1-ccp-10.1177_13591045211048170 – Supplemental Material for Pediatric Avoidant-Restrictive Food Intake Disorder and gastrointestinal-related Somatic Symptom Disorders: Overlap in clinical presentation [file sj-pdf-1-ccp-10.1177_13591045211048170.pdf]

Further information regarding the identification of potentially eligible medical charts.

To identify individuals who met inclusion criteria, a list of all individuals who were admitted to inpatient care in the Eating Disorders Program or other inpatient programs at ***[name of children's hospital removed for blinded review]*** who had a discharge diagnosis (most responsible diagnosis, comorbid diagnosis, or secondary diagnosis) of "other eating disorder", "somatization disorder" or "conversion/functional neurological symptom disorder" based on ICD codes in the Discharge Abstract Database were obtained (n = 397 inpatients). Medical records of all individuals who were seen in the ***[name of eating disorder program removed for blinded review]*** (*n*=525 outpatients and day treatment) were also reviewed to identify those with a diagnosis of ARFID. Furthermore, medical records of individuals who had a predominant GI symptom and were assessed for and/or participated in the ***[name of SSRD treatment program removed for blinded review]*** (*n*=15) were reviewed to identify those who would meet criteria for the present study.

Further information regarding the diagnostic categorization of included patients.

For some patients, the diagnosis was extrapolated from available information (e.g., patients were categorized as having an ARFID diagnosis for the present analysis if they presented with an unspecified eating disorder with a provisional diagnosis of ARFID, and patients who had a different eating disorder diagnosis at assessment where the diagnosis changed to ARFID during treatment). For patients who were identified as having both diagnoses, two members of the research team (clinical psychologists with expertise in eating disorders ***[author initials removed for blinded review]*** and SSRDs ***[author initials***

removed for blinded review]) reviewed the available information on diagnosis and treatment plan to determine the most responsible diagnosis.
